# Supplementary material for: Evidence of ghost plagioclase signature induced by kinetic fractionation of europium in the Earth’s mantle
Source: Nat Commun. 2023 Feb 25;14:1099. doi: 10.1038/s41467-023-36753-0 (PMC9968321; doi:10.1038/s41467-023-36753-0)
Supplement: Supplementary file 1 — Supplementary Information [file 41467_2023_36753_MOESM1_ESM.docx]

**Sample details**

The Oran volcanic field in northwest Algeria is dominated by Miocene to Plio-Pleistocene calc-alkaline and alkaline volcanic rocks, which contain a large number of mantle xenoliths [^1, and references therein^](#_ENREF_1). The xenoliths are very fresh and typically 5–7 cm in diameter. Most samples show a modal compositional trend from fertile lherzolite to olivine-rich harzburgite; refractory lithologies such as olivine-rich lherzolite and harzburgite are the most abundant. A small population (<10 % of the xenoliths) is characterized by anomalously high clinopyroxene contents and was classified by [Hidas, Garrido ^1^](#_ENREF_1) as clinopyroxene-rich lherzolites and wehrlites. Such lithologies have been interpreted in the same volcanic field by [Marchesi, Konc ^2^](#_ENREF_2) as the product of interaction between SiO_2_-undersaturated magmas similar to the host alkali basalts; they are not included in this study. Most xenoliths are from the spinel fa; rare plagioclase occurs (typically <0.5 %) in a locality of the Aïn Témouchent massif.

We selected for this study 12 samples of spinel-facies lherzolites and harzburgites (and a dunite) representative of the textural and lithological diversity among the xenoliths of the Oran volcanic field. Samples with label SOU have been collected in the 2.1-Ma lava flows at the Oued Abdellah beach of Souahlia (35º5’45.58’’N, 1º52′’33.23’’W), whereas those with labels DZ (Djebel Dzioua; 35º17’21.36’’N, 1º14’43.71’’W), GU (Djebel Gueriane; 35º11’28.80’’N, 1º12’ 27.00’’W) and HAM (Hammar E’Zohra; 35º15’42.81’’N, 1º8’41.65’’W) are from the 1.3–0.8-Ma basaltic flows in the Aïn Témouchent massif. A more detailed description of the xenoliths is provided by [Hidas, Garrido ^1^](#_ENREF_1).

**
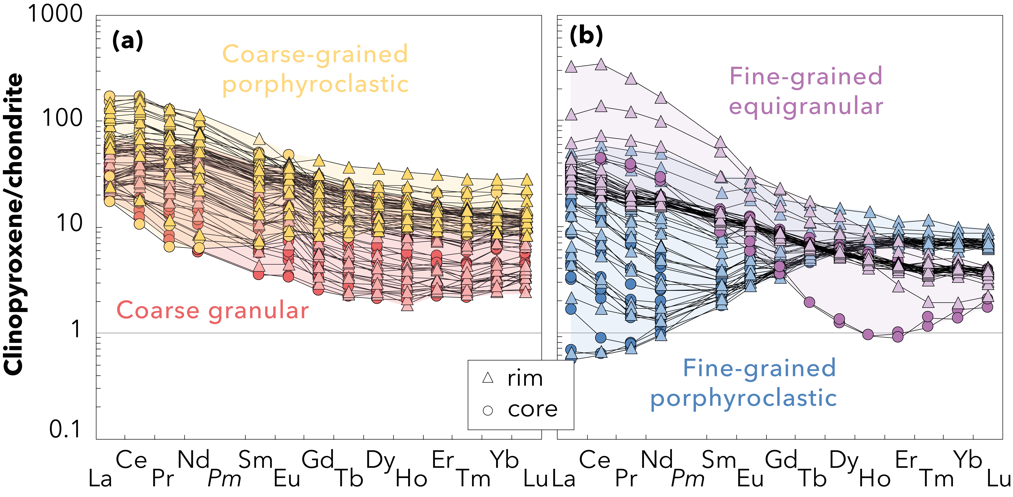
**

**Figure S1. Chondrite-normalized REE compositions in clinopyroxene.** The different textural groups of peridotites defining the compositional field in Fig. 1, 2 & 4 are shown.

**
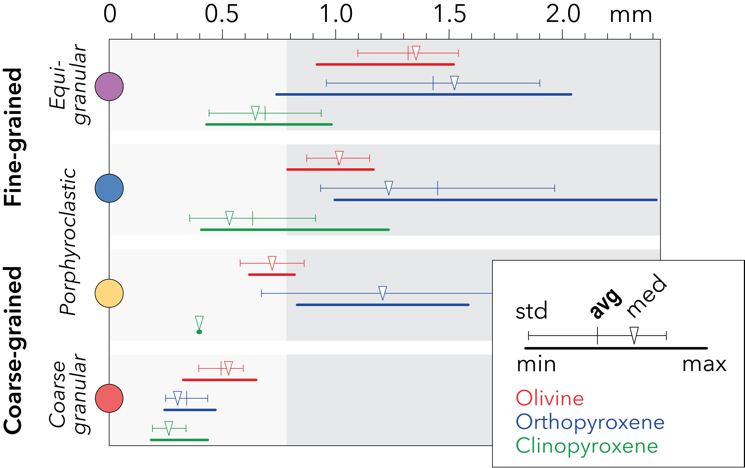
**

**Figure S2. Grain-size distribution.** Olivine, orthopyroxene and clinopyroxene are shown for the different textural groups of peridotites colour-coded as in Fig. 1, 2 & 4.

**Table S1.** Model parameters and inputs.

| **Percolation parameters** | |  |  | |  | |  | |  | |  | |
| --- | --- | --- | --- | --- | --- | --- | --- | --- | --- | --- | --- | --- |
| Melt proportion (porosity) | | 1 % | |  | |  | |  | |  | |  |
| Relative melt velocity (m·s^-1^) | | 1.6·10^−9^ | |  | |  | |  | |  | |  |
| Grain size (mm) |  | 0.01 / 1 | | | |  | |  | |  | |  |
|  |  |  |  | |  | |  | |  | |  | |
| **Partition coefficients** | |  | **Diffusivities** | | | |  | | **Starting compositions** | | | |
|  |  |  | D_0_ | | ε | |  | | Peridotite | | Melt | |
|  |  |  | (m^2^·s^-1^) | | (kJ·mol^-1^) | |  | | (μg·g^-1^) | | (μg·g^-1^) | |
| **La** | 0.044 |  | 4.20·10^−3^ | | 519300 | |  | | 0.56 | | 855.8 | |
| **Ce** | 0.088 |  | 2.20·10^−3^ | | 508200 | |  | | 0.62 | | 652.5 | |
| **Pr** | 0.128 |  | 1.08·10^−3^ | | 495702 | |  | | 0.71 | | 412.1 | |
| **Nd** | 0.188 |  | 5.54·10^−4^ | | 483900 | |  | | 0.95 | | 260.3 | |
| **Sm** | 0.308 |  | 1.42·10^−4^ | | 460100 | |  | | 1.94 | | 94.8 | |
| **Eu^3+^** | 0.364 |  | 7.79·10^−5^ | | 449602 | |  | | 2.77 | | 63.9 | |
| **Eu^2+^** |  |  | 1.20·10^−1^ | | 510448 | |  | |  |  |  |  |
| **Gd** | 0.417 |  | 4.04·10^−5^ | | 438077 | |  | | 3.97 | | 43.1 | |
| **Tb** | 0.458 |  | 2.09·10^−5^ | | 426552 | |  | | 5.04 | | 35.6 | |
| **Dy** | 0.503 |  | 9.97·10^−6^ | | 413600 | |  | | 5.98 | | 29.8 | |
| **Ho** | 0.523 |  | 5.61·10^−6^ | | 403502 | |  | | 6.47 | | 26.0 | |
| **Er** | 0.544 |  | 2.73·10^−6^ | | 390900 | |  | | 6.74 | | 23.5 | |
| **Tm** | 0.544 |  | 1.50·10^−6^ | | 380452 | |  | | 6.88 | | 21.1 | |
| **Yb** | 0.545 |  | 8.83·10^−7^ | | 371200 | |  | | 7.02 | | 19.2 | |
| **Lu** | 0.535 |  | 4.03·10^−7^ | | 357402 | |  | | 7.02 | | 17.5 | |
| REE^3+^ diffusivities are calculated using a constant activation volume V = 10^-5^ m^3^·mol^-1^. | | | | | | | | | | | |  |

**Supplementary references**

1. Hidas K*, et al.* Lithosphere tearing along STEP faults and synkinematic formation of lherzolite and wehrlite in the shallow subcontinental mantle. *Solid Earth* **10**, 1099–1121 (2019).

2. Marchesi C*, et al.* Multi-stage evolution of the lithospheric mantle beneath the westernmost Mediterranean: Geochemical constraints from peridotite xenoliths in the eastern Betic Cordillera (SE Spain). *Lithos* **276**, 75-89 (2017).
